# Decision criteria for the selection of analytical instruments used in clinical chemistry: III Non–monetary criteria

**DOI:** 10.1155/S1463924680000090

**Published:** 1980

**Authors:** H. Büttner

**Affiliations:** Institute for Clinical Chemistry Medical School Hannover Germany


### Decision criteria for the selection of analytical instruments used in clinical chemistry

### III Non-monetary criteria

**H. Büttner**
*Institute for Clinical Chemistry, Medical School, Hannover, West Germany.*

THE selection of analytical instruments is a very complex problem for a clinical chemical laboratory. The reason for this is that medical, methodological and practical organisational aspects must all be considered simultaneously. It is necessary to develop criteria for these aspects which relate to characteristic and relevant features of the instruments concerned, which can be verified, and which may be quantified, wherever possible.

At present, there is no consensus agreement on criteria of this kind, but there are, however, some recommendations from international scientific associations relating to particular problem areas [1,2].

After the objectives have been defined as already outlined, the examination of the data for selection should be carried out in the following order of priority:
(1) Applicability to the intended purpose.
(2) Performance of the instrument.
(3) Practicability.

### Applicability to the intended purpose

Clinical chemical analyses are not only required for diagnostic but also for prognostic purposes, for follow-up and above all, for decisions relating to therapy. There is therefore a wide range of requirements which the analytical instruments must satisfy. These are listed in Table 1.

**Table 1: Decision criteria**

Single or multiple testing
- fixed program
- possibility of selecting tests according to request
- possibility of selecting clinically orientated groups of tests

Analytical mode
-analytical principle (photometric, others)
- endpoint or reaction rate measurement

Sample size (volume)
- use for paediatric departments

Degree of mechanisation
- expected number of patient specimens
- fully mechanised (or automated) system needed
- electronic data processing needed

Possibility of performing statistical emergency tests

The requirements will also vary with the type of hospital to be served, i.e. whether general, small, paediatric, intensive care unit, etc.

Frequently, instrument manufacturers claim 'universal' applicability as a significant advantage of their analytical instruments. However, practical experience shows that this is rarely the case; a simple instrument designed for a specific purpose is often to be preferred to a more sophisticated so-called 'universal' instrument.

### Performance of the instrument

Once the type of analytical instrument has been determined using the clinical requirements to provide the basic data, suitable criteria must be applied to determine the performance of the individual instrument. Examples of these criteria are shown in Table 2.

**Table 2: Decision criteria for instrument performance**

Analytical reliability of the measuring part of the instrument
- precision
- accuracy
- specificity
- detection limit

Speed criteria
- specimens/samples per time unit (frequency)
- analysis time
- throughput time

Correction of interferences
- automated detection and correction of interferences such as two wave lengths' turbidity

Contamination
- carry-over between samples

Stability
- temperature
- measurement process (drift, noise)

The analytical reliability is a predominant feature, and for the assessment of this element suitable criteria already exist. The factors to be considered are precision and accuracy and detection limit, both for the final measuring elements of the instrument and for clinical chemical techniques of analysis used on the instrument. The testing protocols of, for example, the recommendations of the IFCC Expert Panel on Quality Control (Approved Recommendation (1978) on Quality Control in Clinical Chemistry; part 2. Assessment of Analytical Methods for Routine Use) may be used in the appropriate context to obtain comparable data.

At present no universally acceptable testing protocols have been established for the other criteria mentioned.

### Practicability of the instrument

Under this heading it is necessary to take into consideration the specific requirements of the laboratory concerned, including the number of suitable personnel available, their qualifications, the space allocated, financial resources

**Table 3: Decision criteria for the practicability of the instrument**

| | |
|---|---|
| (1) | Space required |
| (2) | Energy consumption, other services required |
| (3) | Ease of operation<br>Operation by unskilled personnel |
| (4) | Adaptability and changeover of analysis methods |
| (5) | Possibility of introducing urgent samples into routine procedures |
| (6) | No restrictions in terms of selection of reagents which can be used |
| (7) | Safe operation |
| (8) | Fault detection and signalling, e.g. using microprocessors |
| (9) | Easy maintenance |
| (10) | Operating instructions |
| (11) | Safety for the operating personnel |
| (12) | Environmental aspects, e.g. production of effluent, waste |

available, and many other factors. The list in Table 3 is by no means exhaustive and has been designed to facilitate the decision as to whether a given instrument is practicable under the conditions prevailing in the laboratory concerned.

These criteria cannot be assessed as objectively as those given previously.

### Conclusions

Consideration of the above criteria should make it possible to arrive at an objective decision at all times. However, it is essential that the following conditions are satisfied: (1) that suitable, quantifiable test parameters exist for these criteria; (2) that requirements are laid down for these test parameters, e.g. optimum values; and (3) that suitable data and information are available to cover the range of instruments from which selection is to be made. The conditions are far from satisfied and for this reason the IFCC Expert Panel on Instrumentation has a most important task – the evaluation and drafting of appropriate recommendations and standards.

REFERENCES

1. Commission on Automation, IUPAC Section of Clinical Chemistry. Characteristics and attributes of instruments intended for automated analysis in clinical chemistry. IUPAC Information Bulletin 1978, No. 3, 234-240.

2a. Büttner, J., Borth, R., Boutwell, J. H., and Broughton, P. M. G. (1978 b) Approved Recommendation on Quality Control in Clinical Chemistry. Part 1. General Principles and Terminology. Clin, Chim. Acta, to be published. Provisional Recommendation was published Clin. Chim. Acta **63**, F25 - F38 (1975).

b. Buttner, J., Borth, R., Boutwell, J. H., Broughton, P. M. G., and Bowyer, R. C. (1978 c). Approved Recommendation on Quality Control in Clinical Chemistry. Part 2. Assessment of Analytical Methods for Routine Use. Clin. Chim. Acta, to be published. Provisional Recommdendation was published Clin. Chim. Acta **69**, F1 - F7 (1976).

c. Buttner, J., Borth, R., Boutwell, J. H., Broughton, P. M. G., and Bowyer, R. C. (1977) Provisional Recommendation on Quality Control in Clinical Chemistry. Part 3. Calibration and Control Materials. Clin. Chim. Acta **75**, F11 - F20.

d. Buttner, J., Borth, R., Boutwell, J. H., Broughton, P. M. G., and Bowyer, R. C. (1978 a) Provisional Recommendation on Quality Control in Clinical Chemistry. Part 5. External Quality Control. Clin. Chim. Acta **83**, 191F -202F.

e. Buttner, J., Borth, R., Boutwell, J. H., Broughton, P. M. G., and Bowyer, R. C. (1977) Provisional Recommendation on Quality Control in Clinical Chemistry. Part 6. Quality Requirements from the Point of View of Health Care. Clin. Chim. Acta **74**, F1 - F9.

### Decision criteria for the selection of analytical instruments used in clinical chemistry

### IV External and internal evaluation of analytical instruments in clinical laboratory sciences

**M. Hjelm**

*Department of Clinical Chemistry, University Hospital, DK-5000 Odense, Denmark*

**and T.D.Geary**

*Division of Clinical Chemistry, Institute of Medical and Veterinary Science, Adelaide, South Australia*

THE performance and final choice of an analytical instrument are usually judged on two criteria, external and internal. The former are dependent on the experience of others, especially if the evaluation was carried out under conditions comparable to those in the purchaser's own laboratory. The latter relate to the purchaser's own assessment.

### The clinical requirements

There are, as yet, no "objective" rules for establishing clinical performance criteria for a particular biomedical analysis. Instead, it is frequently necessary to use allowable limits of error which are based upon present knowledge of the clinical requirements for systematic and random errors, while awaiting more accurate data arising from conferences. That held at Aspen in 1976 by the College of American Pathologists [1] forms a suitable guideline for these.

### External evaluations

These may take the form of published papers, reports given at regional, national and international meetings, or documents produced on behalf of or by various national, professional and governmental organisations. This information may be available from the manufacturer, but a list of evaluations has been published in the British Association of Clinical Biochemists' Newsletter, [2] and a revised list in a WHO Newsletter. It is the intention of the IFCC Expert Panel on Instrumentation to publish a revised list on a regular basis.

The Instrumentation Commission of the Clinical Biology Society of France produced a multi-centre evaluation protocol with which they assessed various enzyme rate analysers. This was based upon the National Comittee of Clinical Laboratory Standards Proposed Standard Evaluation Protocol - [1,3] and work by Broughton et al [4]. A working group of the German Society for Clinical Chemistry has taken this document and on behalf of the Expert Panel on Instrumentation of the IFCC, intend to prepare an evaluation procedure which should either be, or provide the basis for, an international standard. The final report prepared from data supplied by more than one laboratory will be less affected by the work patterns of each testing laboratory. It will therefore provide a strong basis for comparison.

#### Multi-centre trial

The proposed procedure will take the form of a multi-centre

